# Repositioning *N*-Acetylcysteine (NAC): NAC-Loaded Electrospun Drug Delivery Scaffolding for Potential Neural Tissue Engineering Application

**DOI:** 10.3390/pharmaceutics12100934

**Published:** 2020-09-30

**Authors:** Gillian D. Mahumane, Pradeep Kumar, Viness Pillay, Yahya E. Choonara

**Affiliations:** Wits Advanced Drug Delivery Platform Research Unit, Department of Pharmacy and Pharmacology, School of Therapeutic Science, Faculty of Health Sciences, University of the Witwatersrand, 7 York Road, Parktown, Johannesburg 2193, South Africa; gillian.mahumane@wits.ac.za (G.D.M.); pradeep.kumar@wits.ac.za (P.K.); viness.pillay@wits.ac.za (V.P.)

**Keywords:** nanofibers, electrospinning, drug delivery, N-acetylcysteine, scaffold, drug release, Poly(D,L-lactide-co-glycolide (PLGA)

## Abstract

Traumatic brain injury (TBI) presents a serious challenge for modern medicine due to the poor regenerative capabilities of the brain, complex pathophysiology, and lack of effective treatment for TBI to date. Tissue-engineered scaffolds have shown some experimental success in vivo; unfortunately, none have yielded consummate results of clinical efficacy. N-acetylcysteine has shown neuroprotective potential. To this end, we developed a N-acetylcysteine (NAC)-loaded poly(lactic-co-glycolic acid) (PLGA) electrospun system for potential neural tissue application for TBI. Scanning electron microscopy showed nanofiber diameters ranging 72–542 nm and 124–592 nm for NAC-free and NAC-loaded PLGA nanofibers, respectively. NAC loading was obtained at 28%, and drug entrapment efficacy was obtained at 84%. A biphasic NAC release pattern that featured an initial burst release (13.9%) stage and a later sustained release stage was noted, thus enabling the prolonged replenishing of NAC and drastically improving cell viability and proliferation. This was evidenced by a significantly higher cell viability and proliferation on NAC-loaded nanofibers for rat pheochromocytoma (PC12) and human glioblastoma multiform (A172) cell lines in comparison to PLGA-only nanofibers. The increased cell viability and cell proliferation on NAC-loaded nanofiber substantiates for the repositioning of NAC as a pharmacological agent in neural tissue regeneration applications.

## 1. Introduction

Traumatic brain injury (TBI) is the greatest contributor to global death and disability among all trauma-related injuries. The global incidence of traumatic brain injury (TBI) is generally reported as ~200 in 100,000 with a mortality of 20 per 100,000 people [1]. Although common, the impact of neurotrauma is difficult to describe and may be underestimated due to the variable way statistics are recorded; trauma registries are poor (even in developed countries), and most figures are based on admission and do not include pre-hospital deaths, which account for ≈50% of road trauma deaths [1,2]. Therefore, traumatic brain injury is a silent global epidemic for which surveillance and interventions are sorely lacking (therapies, funding, dedicated research centers, and human resource).

This is in part due to the complex evolutionary pathophysiology of TBI. Complete neurological damage does not all occur immediately at the moment of primary injury. Secondary injury (follows within hours of primary TBI) has been identified as the leading cause of in-hospital deaths post TBI [3]. An estimated 80 to 90% of the patients who die reportedly show evidence of ischemic brain damage in histopathological examinations [4]. This secondary injury is a result of brain swelling, leading to a decreased brain perfusion and thus ischemia, which ultimately contributes to the observed brain damage and ultimate morbidity in some patients. The innate inflammatory response induced by injury is crucial to maintain homeostasis; however, if left unbridled into a chronic state, this leads to oxidative stress and glial scarring [5,6]. The glial scar tissue not only secretes inhibitory molecules (e.g., chondroitin sulphate proteoglycans (CSPGs) and myelin-associated glycoproteins), it further creates a mechanical barrier that hinders axonal regrowth [7,8].

Current treatment approaches for TBI are largely focused on preventing further damage (pharmacological agents and rehabilitation) instead of facilitating regeneration [9]. Several pharmacological agents such as free-radical scavengers and calcium channel blockers have been investigated to prevent the secondary injury cascade associated with TBI, to no avail [10].

Tissue engineering aims to engender scaffolds based on biological systems, particularly, the nano–micro–macro extracellular matrix environment of the cell to aid in tissue repair and regeneration. Biological systems constitute a myriad of nanoscale structures, and the benefits of the nanotherapies have been widely reported in the literature [11]. In particular, the neuron has been likened to a nanofiber with the fundamental function to receive, conduct, and transmit neurochemical-induced signals [12]. Therefore, several approaches have been used for the design of nanoscale scaffolds for neurotherapies, and many prototypes are based on well-established knowledge of the brain extracellular matrix (ECM). The ECM has been described as an interwoven network of nanofibers embedded in a hydrated gel-like matrix [6,13] with the ECM cells (astrocytes, neurons, oligodendrocytes, and microglia) constituting the framework of the milieu at the nanometer scale (viz. proteinaceous collagen fibrils ranging between 50 and 200 nm and fibronectin fibrils of 2–3 nm thick and 60–70 nm long) [6,14] that facilitate interaction with the ECM [13]. Therefore, it follows logic to design biomimetic scaffolds with precise nanoscale features to provide pseudo-in vivo architectural cues to optimally restore cell-cell interactions for neural (brain) tissue engineering applications. 

Tissue-engineered biomaterial scaffolds have shown various degrees of experimental success [15]. For instance, Zhou et al., showed that cells could adhere better on a mesenchymal stem cell (MSC)–PLGA scaffold complex than the PLGA with improved to cell adhesion and migration in vivo 14 days after TBI [16]. Yang et al. observed that the rate of neural stem cell differentiation was higher for poly(l-lactic acid (PLLA) nanofibers than that of micro fibers, independent of nanofiber alignment [17]. Unfortunately, there is yet to be a 3D scaffold that exhibits consummate results of an effective clinical standard to date [15].

This study focused on the design of electrospun PLGA nanofibers based on a clinical pharmacological drug, creating a localized therapeutic signal within the nanofibers for brain tissue engineering with optimal porosity, superior surface area to volume ratio, and cell viability and proliferation biocompatibility, which were fabricated from biocompatible materials in order to improve localized drug delivery at the brain lesion site.

The therapeutic focus of the current study will be *N*-acetylcysteine (NAC). *N*-acetylcysteine is commonly used to loosen thick mucus in patients with chronic obstructive pulmonary disease (COPD) or cystic fibrosis and an antidote to acetaminophen and carbon monoxide poisoning [18,19]. Several preclinical models of CNS injury have reported on the neuroprotective properties of NAC to control inflammation and ischemia [20,21,22,23,24,25,26]. For instance, Khan et al. reported that the administration of NAC after ischemia onset protected the brain tissue from free radical injury, inflammation, and apoptosis in a rat model of experimental stroke [26]. Xiong et al. reported that NAC could restore brain glutathione levels and mitochondrial glutathione levels after TBI [27]. Although NAC crossed the blood–brain barrier (BBB) [22,25], its potential neurotherapeutic effects have been largely limited by multiple factors. NAC has a low oral bioavailability estimated at 6–10% as a result of extensive first-pass metabolism [28,29,30,31]. In addition, when administered intravenously, systemically, NAC binds to plasma proteins, and about 30% undergoes renal elimination resulting in a short half-life [28]. In addition, increasing systemic doses of NAC for superior (passive) transport of NAC across the BBB may result in increasing the blood pressure [31]. Incorporating the NAC in a biocompatible substrate will enable localized delivery of the NAC at the target site, bypassing its limitations from alternate administrations routes (oral, intravenous) and thereby enhancing its therapeutic effect. 

We used poly(d-l-lactide-*co*-glycolide) (PLGA) as the substrate to embed and deliver the therapeutic agent (NAC). Poly(d-l-lactide-*co*-glycolide) (PLGA) nanofibers have been explored as desirable biomaterials for nanoscale electrospinning in neural tissue engineering [32,33,34]. The available flexibility in its polylactic acid (PLA) and poly glycolic acid (PGA) ratios enable the precise control of scaffold properties (e.g., mechanical strength, biotensile properties, biodegradation, bioactive release kinetics, porosity, neuro-durability, anti-fouling) for neural tissue engineering [35].

Therefore, we hypothesize that embedding NAC within the electrospun PLGA nanofibers as a neuro-insert will overcome the extensive plasma protein binding observed after oral delivery and will also provide preferential spatial delivery of NAC to the injured brain tissue to ensure neuroavailability [36] and optimal neuroprotective effects within a neuromimetic scaffold. In order to control the release of the low molecular weight and hydrophilic NAC from the nanofibers inserted at the TBI site, PLGA with a 85:15 PLA/PGA polymer ratio was selected as the ideal polymer platform for nanofiber synthesis. The NAC-embedded PLGA nanofibers were electrospun into a neuromimetic scaffold and fully characterized to determine its potential for neural tissue engineering in TBI. Several pharmaceutical stability and chemical integrity experiments were undertaken to support the function of the nanosystem via Fourier Transform Infrared (FTIR), TGA, DSC, SEM, BET, physicomechanical analysis, contact angle, XRD, and NAC release kinetics as reported herein. In addition, ex vivo cytotoxicity studies were undertaken on PC12 and A172 cell lines to assess the neurocompatibility of the nanosystem.

## 2. Materials and Methods 

### 2.1. Materials

Poly(d,l-lactide-coglycolide Resomer RG 858 S(PLGA) 85:15 (PLGA) was supplied Ex gratia from Evonik Nutrition & Care GmbH, Essen, Germany. This particular PLGA Resomer 858 used herein is a bioresorbable poly(d,l-lactide-*co*-glycolide) (85:15) grade suitable for pharmaceutical applications such as controlled release and acting as a long acting medication delivery material. *N*-acetylcysteine (NAC), 2,2,2-trifluoroethanol (≥ 99% GC), formic acid, Dulbecco’s Modified Eagles Medium—high glucose, Roche Cell Proliferation Kit I (MTT), dimethyl sulfoxide (DMSO), and trypsin–EDTA solution were obtained from Sigma Aldrich (Steinheim, Germany). Donor Equine Serum (DES) was purchased from Hyclone. Ethanol (99% absolute) was purchased from LabChem, Edenvale, Johannesburg, South Africa. Gibco Fetal Bovine Serum and Ham’s F-12 Nutrient Mix (F12) were purchased from Thermofischer Scientific (Germiston, Gauteng, South Africa). Rat (Rattus Norvegicus) adrenal gland pheochromocytoma PC12 and human glioblastoma multiforme A172 cells were purchased from Cellonex (Separations, South Africa). Pencillin/Streptomycin/Amphotericin B (P/S/AB) solution was purchased from Lonza, Oregon, USA.

### 2.2. Fabrication of the NAC-Loaded Electrospun System 

The electrospun systems were prepared via the electrospinning technique using the NanoSpinner24 (Inovenso Ltd., Co., Istanbul, Turkey). A 1:1 ratio comprising 2,2,2-trifluoroethanol/formic acid (TFE/FA) electrospinning solvent mixture was prepared. The PLGA and NAC (one part NAC to two parts PLGA) were added to the TFE/FA solvent mixture at room temperature and stirred under magnetic stirring for 2 h. This NAC/PLGA ratio enabled the maximal NAC amount while maintaining electrospinnability of the solution (no erratic fiber deposition, beading, or precipitation of drug particles out of the solution). Then, the solutions were transferred into 10 mL syringes, and a pump was used to dispense the solution through a spinneret (0.8 mm) at a controlled rate of 300 µL/hr. The applied voltage was controlled between 24 and 26 kV. The distance between the spinneret tip and the aluminum-clad rotating cylindrical collector was set at 200 mm with the collector rotating at 400 rpm and a stroke velocity of 200. The pure PLGA electrospun scaffold was subsequently used as the control. Electrospinning experiments were performed at room temperature under the relative humidity of ≤ 40%. The resultant nanofibers were subsequently collected and vacuum dried for 24 h at room temperature for the removal of any residual solvent.

### 2.3. Characterization of the NAC-Loaded Electrospun System

These processes could lead to significant changes on the polymer and crystal structure of the incorporated NAC. Therefore, FTIR, TGA, DSC, and XRD studies were performed in order to confirm the presence of the NAC in the electrospun fibers and to evaluate the structural changes in the polymer matrix and NAC crystallinity after the electrospinning process. The chemical transitions of the electrospun nanofibers before and after NAC loading were determined using a Fourier Transform Infrared (FTIR) spectrometer (PerkinElmer Spectrum 100, FT-IR Spectrometer, Beconfield, UK) fitted with a universal ATR Polarization Accessory (PerkinElmer Ltd., Beconfield, UK). The samples were analyzed at high resolution over a 4000–650 cm^−1^ wavelength range on a Nicolet Impact 400D FTIR Spectrophotometer coupled with Omnic FTIR research grade software (Nicolet Instrument Corp., Madiso, WI, USA). The presence/absence of specific functional groups was noted and analyzed. Systems need to be stable at the physiological tissue target temperatures in vivo. 

Effects of NAC-loading on thermal stability were characterized using thermogravimetic analysis (Perkin Elmer, TGA 400, Waltham, MA, USA). Samples (10–15 mg) were placed in an unsealed ceramic pan under nitrogen atmosphere at a flow rate of 20 mL.min^−1^. Thermograms were recorded in the temperature range of 30–600 °C with a heating rate of 10 °C/min, revealing the mass changes with increasing temperature, thermal stability, and degradation temperature of the sample material.

The effects of NAC-loading on thermal transitions was characterized using differential scanning calorimetry (DSC) (Mettler Toledo, STARe System, Schwerzernback, Zurich, Switzerland). Dried samples (5–10 mg) were weighed into aluminum crucible pans (40 mL) and sealed shut. Thereafter, samples were heated at a temperature range of 10–300 °C at a rate of 10 °C·min^−1^. Heating took place under nitrogen atmosphere (Afrox, Germiston, Gauteng, South Africa) at a flow rate of 200 mL·min^−1^, acting as the purge gas to reduce oxidation. The thermal profiles were analyzed. 

Amorphization of NAC will contribute to improved solubility and increased NAC at the target site. X-ray diffraction (XRD) studies were performed using an X-ray diffractometer (Rigaku Miniflex 600, Rigaku Corporation, Matsubara-cho, Akishima-shi, Tokyo, Japan) to obtain information about the crystalline or amorphous state of NAC after electrospinning. The X-ray diffractograms were attained with the scanning scope of 2*θ* from 3° to 90° at room temperature. XRD was operated at a voltage of 40 kV, a current of 15 mA at a scan step of 0.02°, and a speed of 10°/min. Integrated X-ray Powder Diffraction software (PDXL 2.1, Rigaku, Tokyo, Japan) was employed for sample analysis.

The size and morphological characteristics of all electrospun scaffolds were characterized utilizing scanning electron microscopy (SEM) FEI Nova NanoLab SEM (FEI Company, Hillsboro, OR, USA). Gold isotope and carbon were used to sputter coat the sample using the Micromeritics Porositometer (Micromeritics ASAP 2020, Norcross, GA, USA); surface areas, pore sizes, shapes, and distributions can be measured according to the parameters presented in Table 1 and analyzed with reference to the parameters in Table 2. Adequate mechanical stiffness is necessary to not only provide a suitable micromechanical environment and promote neuronal anchoring but also to avoid injuring the surround soft neural tissue at the lesion site. The mechanical properties of electrospun fiber mats were determined with a texture analyzer (TA.XTplus texture analyser, Stable Microsystems, Surrey, UK) fitted with two clamps. The width, height, and length of the samples were measured with a digital Vernier calliper (Krafft, DV150GW, Schoellerstr Düren, German) having a precision of 1 μm. A rectangular 40 × 10 mm strip of 1 mm thickness was fixed at 20 mm between the two clamps. Tests were conducted at 0.167 mm/s while applying a 0.5 N trigger force.

### 2.4. NAC Loading, Entrapment Efficiency, and NAC Release Kinetics

Scaffold hydrophilicity can impact the NAC release rate; therefore, the hydrophilicity of the nanofiber systems was evaluated using the DataPhysics Instruments contact angle goniometer by measuring the water contact angle using the sessile drop method. The nanofiber mats were placed on the testing plate and kept smooth. Subsequently, 2 µL of distilled water was dispensed onto the surface at a dosing rate of 2 µL/s using a Hamilton syringe. The images of the water droplet were recorded by camera software after the droplet was stable. Thereafter, the water contact angle was measured using the SCA202 version 4.1.12 build 1019 software. Five different samples were measured, and the average values were calculated. Drug entrapment efficiency, drug loading, and cumulative NAC release from the scaffolds was quantified by WinASPECT^®^ Spectro analytical Software (Analytik Jena AG, Jena) through ultraviolet spectroscopy at a wavelength of 224 nm. 

### 2.5. Effect of NAC Loading in Electrospun Nanofiber System on PC12 and A172 Cell Viability and Proliferation In Vitro 

The PC12 cells were cultured in F12 media containing 15% DES, 2.5% FBS, and 1% P/S/AB solution. The A172 cells were cultured in Dulbecco’s Modified Eagles Medium (DMEM) supplemented with 20% FBS and 1% P/S/AB solution. Both cell lines were maintained under 5% CO_2_ atmosphere at 37 °C. Before the test was performed, the nanofibers were cut into 0.5 × 0.5 cm mats and sterilized under UV light for 30 min (UV-C 254 nm, UV Equip, Johannesburg, South Africa). The sterilization of scaffolds is a crucial step relating to the applications for tissue engineering in vitro and ultimately, clinical use of the biomaterial scaffold. Braghirolli and co-workers evaluated the effects of sterilization methods on electrospun PLGA nanofibers and the subsequent adhesion efficacy of mesenchymal stem cells. Their observation noted that other methods of sterilization such as ethanol (70%) or using an antimicrobial solution resulted in morphological and structural alterations to the scaffold, respectively [38]. UV sterilization was noted to generate sterile scaffolds without visible changes in the morphological appearance of the electrospun scaffold. Therefore, UV sterilization was used herein to sterilize the scaffold prior to in vitro evaluation. The PC12 and A172 cells were seeded onto the scaffolds in 96-well plates at a density of 1 × 10^4^ cells per well. Absorbance was measured at 570 nm with a background subtraction at 690 nm using a multi-plate reader (Biotek, Winooski, VT, USA). The absorbance reading of the blank (690 nm) was subtracted from all the samples.

## 3. Results and Discussion

### 3.1. NAC–Polymer Interaction Analysis by Fourier Transform Infrared Spectroscopy (FTIR)

**Figure 1:**
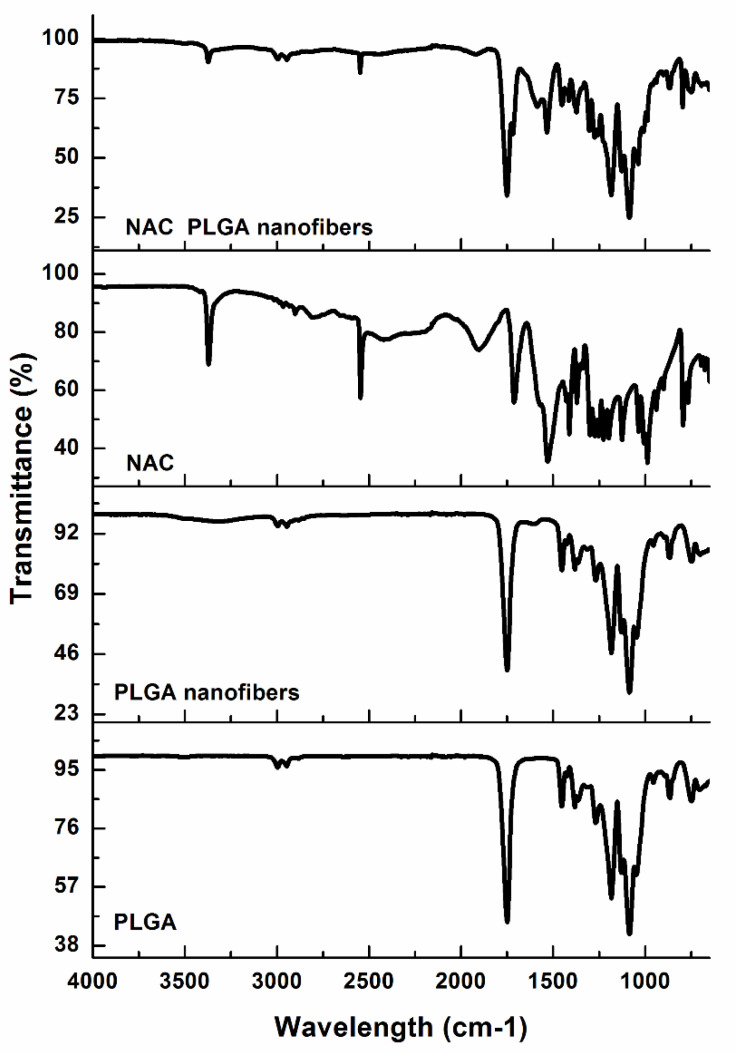
Fourier transform infrared (FTIR) spectra of the native materials (NAC, PLGA), electrospun PLGA and NAC-loaded nanofiber. NAC: N-acetylcysteine, PLGA: poly(lactic-co-glycolic acid).

The FTIR spectra of native PLGA and the PLGA nanofibers characterized by bands in the regions 2995 and 2947 cm^−1^ are present due to the alkyl groups [39,40] and carbonyl stretching around 1749 cm^−1^ and C-O bands in the 1067–1454 cm^−1^ region of both spectra, indicating the presence of the ester group (Figure 1). These groups contribute to the NAC delivery properties of the PLGA nanofiber system. PLGA biodegrades by hydrolysis of its ester linkages in water [41], resulting in PLGA degradation-induced NAC release. Since the hydrophobic portion forms that majority (PLGA 85:15), rapid degradation will be avoided, contributing to a sustained NAC release profile. Comparison of the IR spectra of the native PLGA and PLGA nanofibers revealed that there were no significant changes in the two FTIR spectra. This indicated the molecular stability of PLGA in the electrospinning process during nanofiber formation. In keeping with the literature [42], the FTIR spectrum of NAC showed a narrow N-H stretching band at 3371 cm^−1^ indicative of a free NH and the strong S-H stretching band at 2546 cm^−1^. At lower frequencies, a strong C=O stretching band of the carboxylic group, amide I, II, and III was observed at 1714, 1560, 1530, and 1274–1371 cm^−1^, respectively. Loading NAC in PLGA caused some shielding of some of the NAC functional groups (NAC was in lower quantity to PLGA), which can be used as evidence of success in NAC loading. Moreover, the presence of both NAC and PLGA were discerned in the FTIR spectra of NAC-loaded nanofibers. In comparison to the PLGA nanofiber spectrum, the composite NAC-loaded PLGA spectrum showed additional peaks at 1533 cm^−1^ (amide II), 1606 cm^−1^ (amide I), 1710 cm^−1^ (carboxylic group), 2548 cm^−1^ (S-H), and a low-intensity peak at 3375 cm^−1^ (NH), respectively, indicating the presence of NAC in the nanofibers. 

### 3.2. Analysis of NAC Loading on Thermal Stability of Electrospun System 

**Figure 2:**
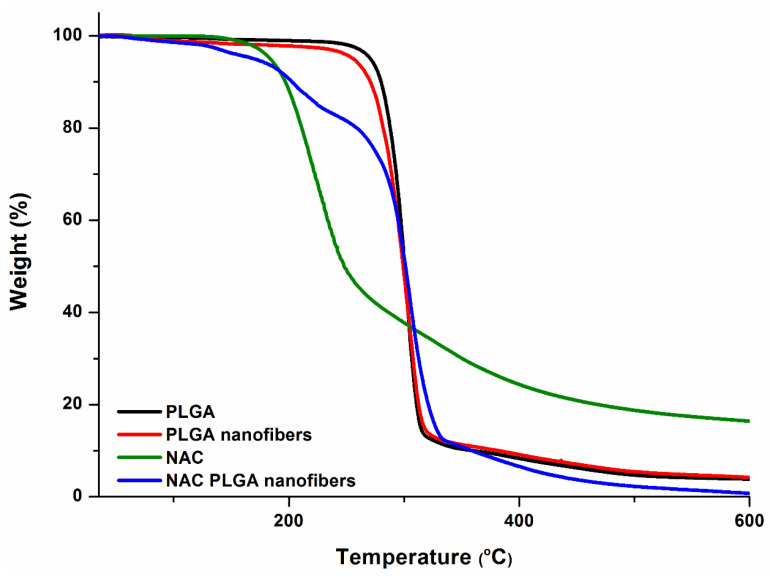
Thermogravimetric curves of PLGA (bulk), PLGA nanofibers, NAC and NAC/PLGA nanofibers.

Thermogravimetric analysis was conducted to evaluate the thermal stability and extension of degradation effects during processing by comparing the native PLGA and NAC and the resultant scaffold (Figure 2). Thermogravimetric analysis also detected the moisture content (water or solvent). The thermograms revealed a weight loss in the curves under 110 °C, which was attributed to the evaporation of volatile materials/moisture content; for all curves, these depressions were <5%. (Figure 2), contributing to greater stability over time. Both native PLGA and PLGA nanofibers presented thermal stability in a lower temperature range (<250 °C) where significant weight loss occurred in the range of 268–340 °C (native PLGA) and 253–330 °C (PLGA nanofibers). The closeness between the respective values of T_onset_ and T_deg.max_ for bulk PLGA and PLGA nanofibers obtained from electrospinning indicates the good preservation of polymer thermal properties during nanofiber fabrication. However, the PLGA nanofibers were more reactive than the native PLGA, in part due to their larger surface area, and consequently, they suffered thermal decomposition more quickly. However, the thermal decomposition T_onset_ was well above the physiological temperatures in vivo. NAC weight loss was observed in the range 170–600 °C, which was attributed to thermal decomposition. Two significant mass loss stages (168–245 °C and 282–362 °C) were observed for the NAC-loaded PLGA nanofibers attributed to the presence of both NAC and PLGA in the nanofibers. This observation was also confirmed by DSC and XRD, reinforcing the idea that NAC exists in both the amorphous and crystalline phases. The second decomposition temperature range of NAC-loaded PLGA nanofibers is similar to that of bulk PLGA, which is indicative of the preservation of polymer (PLGA) thermal properties during nanofiber fabrication. The initial degradation temperature of NAC when loaded in the nanofibers is higher, suggesting an improvement in thermal stability in comparison to the NAC alone in vivo. The thermal degradation temperature for the NAC/PLGA nanofibers is well above the physiological and processing temperature. Therefore, the fabrication process did not induce significant NAC/PLGA losses, and the NAC-loaded nanofibers have potential thermal stability properties at ambient and physiological temperatures.

### 3.3. Evaluation of NAC Distribution and Crystallinity in NAC-Loaded PLGA Nanofibers

**Figure 3:**
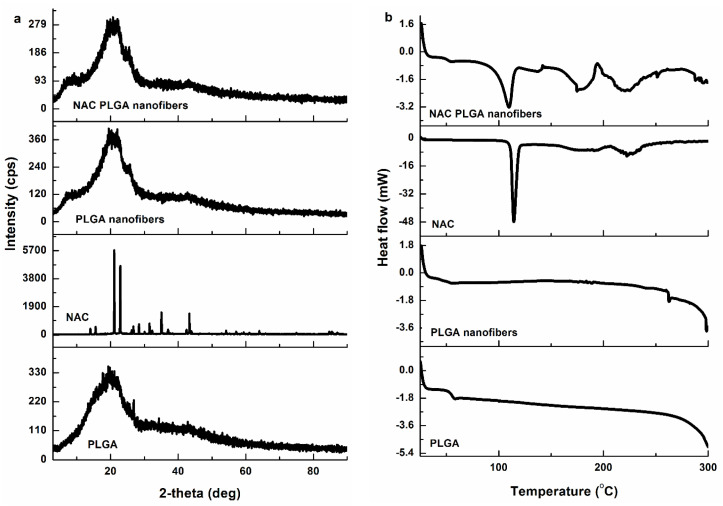
(**a**) X-ray diffraction patterns of PLGA nanofibers, NAC, and NAC/PLGA nanofibers. (**b**) DSC curves of bulk PLGA, PLGA nanofibers, NAC and NAC/PLGA nanofibers.

Drug distribution in the PLGA nanofibers as a function of NAC loading was studied by XRD and DSC analysis. Blend electrospinning was used to fabricate NAC-loaded PLGA nanofibers. With blend electrospinning, the NAC and polymer are present in one solution; this allows the hydrophilic small molecular drug, NAC, to be incorporated not only on the surface of the nanofibers, but it also enables physical entrapment of the NAC inside the polymer matrix of the nanofibers [43]. The X-ray powder diffraction (XRD) patterns showed that the major crystalline diffraction peaks of NAC crystals were observed at the 2θ values of 14.08°, 15.5°, 19.8°, 21.02°, 22.82°, 24.14°, 26.8°, 28.5°, 30.08°, and 32.26° (Figure 3a). 

The NAC-loaded nanofibers displayed a much weaker intensity than that of the PLGA-only nanofibers, indicating a different a lesser degree of crystallinity/ordered structure. Moreover, the diffraction peaks of the NAC crystals were absent in the XRD pattern of NAC-loaded nanofibers, and only the XRD pattern characteristic of the PLGA was observed. These results strongly suggest that electrospinning the NAC/PLGA blend may have resulted in the NAC molecule simultaneously (1) dissolved in the polymer matrix and (2) dispersed in and throughout the nanofiber matrices [44]. This reduced intensity indicates the reduced crystallinity of the NAC, which signified some amorphization. The amorphous phase of PLGA fibers may increase solubility, as the increased surface area enables the NAC to be wet by solvent easier [5,45]. This can help improve the solubility of NAC in the implant site. Although NAC crossed the blood–brain barrier, very little reaches the brain via oral or intravenous routes. This is because NAC has a very low bioavailability due to its binding to plasma proteins in the blood and loss (±0%) through urine [44]. The presence of the NAC crystalline peaks confirms the incorporation of NAC into the nanofibers. The nanofibers can serve as a delivery vehicle to improve NAC delivery to the implant site. The glass transition temperature (T_g_) bulk PLGA and NAC-loaded PLGA nanofibers were observed at approximately 59 and 51 °C, respectively (Figure 3b). The T_g_ of the NAC-loaded PLGA nanofibers was slightly lower than that of the bulk PLGA due to the larger surface area to volume ratio of the electrospun nanofibers as well as the improved orientation of molecular chains [39]. An endothermic peak was noted at 203 °C, which was attributed to the decomposition temperature of PLGA nanofibers [39]. The DSC thermogram of pure NAC showed a sharp endothermic peak that corresponds to melting at 114 °C, indicating its crystalline nature. This crystallinity was further confirmed by the XRD. In addition, an endothermic peak was observed in the NAC/PLGA nanofibers (105 °C) corresponding to NAC. This corroborates the crystalline NAC XRD peaks observed, which suggests that NAC is dissolved in the nanofiber polymer matrix. Thermal decomposition of the NAC peaks was noted at in the range 160–240 °C similar to the observed TGA curves. The T_g_ (glass transition temperature) of the PLGA and NAC/PLGA nanofibers were all above the physiological temperature of 37 °C. Therefore, the nanofibers potentially possess a fairly stable chain structure and enough mechanical strength at ambient and physiological temperatures to be fabricated into thermostable implants/delivery devices.

### 3.4. Morphological Analysis of Electrospun Nanofibers

**Figure 4:**
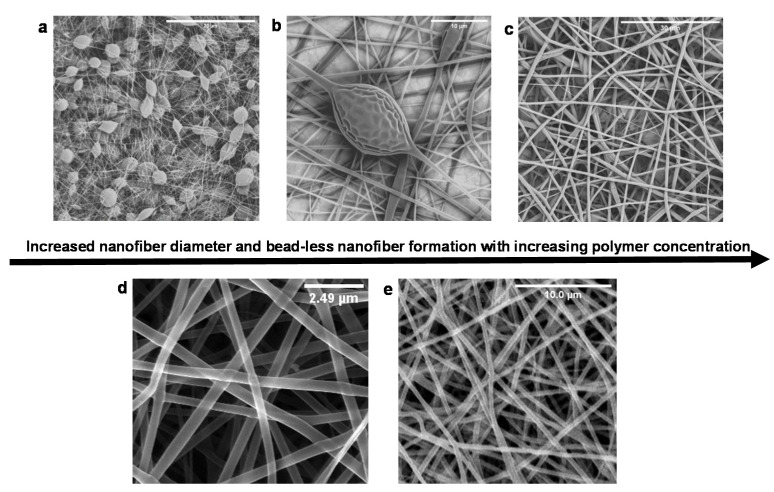
Scanning electron micrograph of electrospun nanofibers. (**a**,**b**) Beaded fibers are formed at low concentrations: (**a**) 10% and (**b**) 15%. (**c**) Bead-less nanofibers were formed at higher concentrations 20%. (**d**): The critical entanglement concentration was noted at 18% PLGA. (**e**) The incorporation of NAC in the nanofibers did not alter nanofiber formation at 18%.

The morphological characteristics, orientation, and diameter distribution of the electrospun nanofibers are shown in the SEM images (Figure 4). The electrospinning process confirmed that for nanofiber formation, a critical entanglement concentration (CEC) was required and determined empirically by screening several candidate polymer concentrations. Below the CEC value (Figure 4a,b), thinner fibers were obtained; however, Rayleigh instability was also observed [46]. Above the CEC value, stable jets were achieved, resulting in the formation of continuous fibers (Figure 4c), which were augmented by the increased polymer chain entanglements. Further increase in the concentration resulted in larger average fiber diameters 1700 ± 416 nm accompanied by a broad diameter distribution (diameter size ranging 254–1733 nm) (Figure 4c). An increase in fiber diameter as a result of increased concentration is a well-noted phenomenon in electrospinning [36,46,47]. Moreover, high concentration presented a challenge to the electrospinning process due to the high viscosity of the polymer solution. This resulted in blocking of the needle tip. At 18% PLGA concentration, stable jets were achieved, resulting in the formation of continuous beadles—randomly aligned nanofibers (Figure 4d,e).

Nanofiber diameters ranged 72–542 nm and 124–592 nm for NAC-free and NAC-loaded PLGA nanofibers, respectively (Figure 4). The average diameter for PLGA nanofibers was 320 ± 128 nm (Figure 4d), whereas the average diameter of NAC-loaded nanofibers was 415 ± 124 nm (Figure 4e). In the native extracellular matrix, randomly oriented collagen fibers range from 50 to 500 nm [48]. The nanofibers produced herein are randomly oriented with interconnected pores. The randomly oriented fibers confer a ‘branching’ effect with intersections creating a net-like structure resulting in porous features (Figure 4a). This is important for achieving cell–cell contact by providing contact guidance and allowing cells to signal processes across multiple fibers. The dimensions of the PLGA nanofibers produced are biomimetic with the native ECM. He and co-workers previously reported 500 nm as the most suitable fiber diameter for a nerve scaffold [49]. The incorporation of NAC led to an increased average fiber diameter. The addition of NAC may have increased the viscosity of the solution, which in turn induced a higher chain entanglement of the polymer, resulting in the observed increase in diameter compared to the NAC-free nanofibers. Regardless, the average NAC-loaded nanofiber diameter (415 nm) produced is within the desired fiber diameter range found in the native ECM 50–500 nm [48].

**Figure 5:**
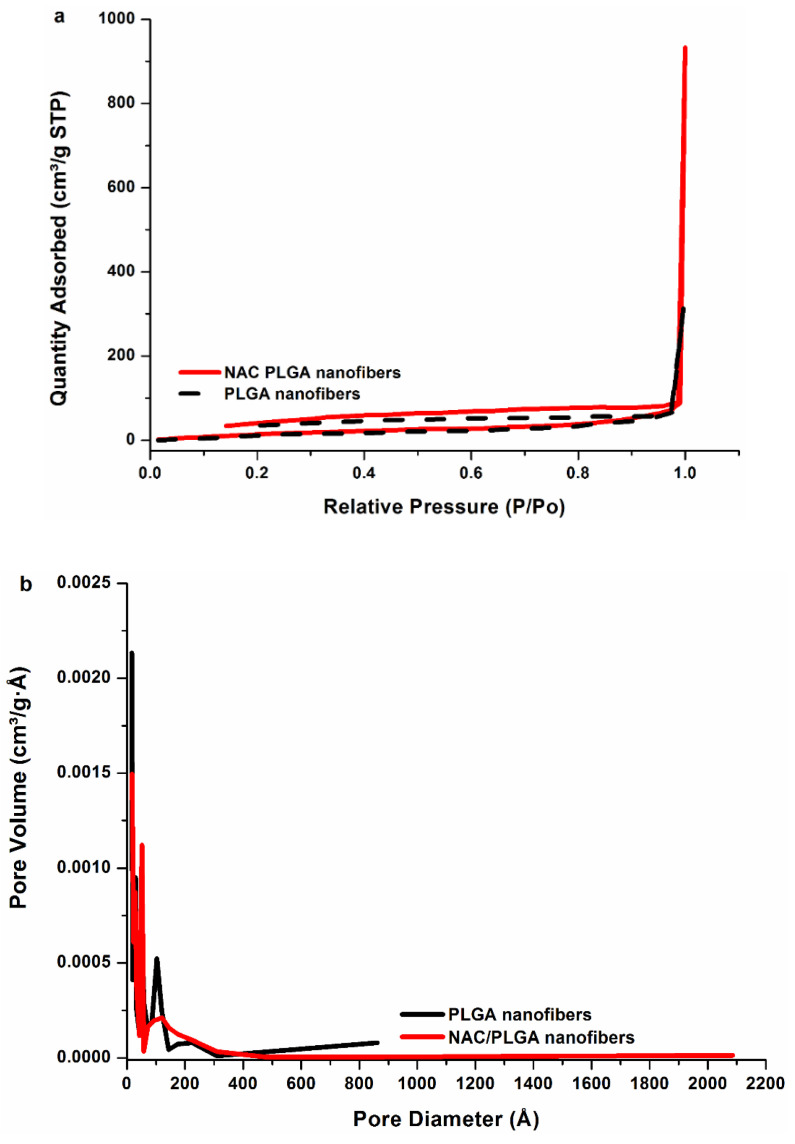
Equilibrium (**a**) adsorption/desorption isotherms and (**b**) pore size distribution of PLGA and NAC/PLGA nanofibers.

The interconnected porous structure is ideal, as it will aid in functioning as a temporary reservoir for the accumulation of the NAC and facilitating the permeability of the nanofibers for uninterrupted passage of cells, nutrients, and waste in the implant site. The high surface area and porous nature of the scaffold was further confirmed by BET analysis. Figure 5 displays the nitrogen adsorption–desorption isotherms of the nanofibers. It was noted that the samples exhibit Type III sorption, confirming the porous nature of the nanofibers. Surface area and porosity were classified according to the criteria stipulated in Table 2. Based on the BET surface area analysis, the specific surface area of the PLGA nanofibers was noted at 401.7019 m²·g^−1^. The high surface area noted for PLGA is attributable to the nanoarchetype that will potentially enhance the surface area for cell–substrate contact and provide a large surface area for cells to adhere in vivo. The lowered NAC/PLGA nanofiber surface area (82.9030 m²·g^−1^) in comparison could be attributed to the larger diameter of the fibers. The isotherm is not closed, which may be due to the swelling phenomenon due to the formation of very narrow slip pores or bottle-shaped pores present over the microporous structure. Nitrogen adsorption below the relative pressure of P/Po < 1 was indicative of the formation of micropores [50]. The nanofibers have a pore size distribution covering both the micro and mesoporous ranges. Figure 5 shows the results for the Barrett, Joyner, and Halenda (BJH) pore size distribution obtained. The plot of pore volume versus pore diameter shows a broad pore size distribution ranging 1.7–86.1 nm for PLGA and 1.7–208.6 nm for PLGA and NAC/PLGA, respectively. Both nanofiber mats presented with similar peak values in the regions 1.7, 2.9, 5.1, 10, and a broad peak in the region 13–25 nm. This is pore size distribution may be a result of the layered and intersecting arrangement of nanofibers consistent with the SEM imaging (Figure 4).

### 3.5. Mechanical Characterization of NAC-Loaded Nanofibers 

**Figure 6:**
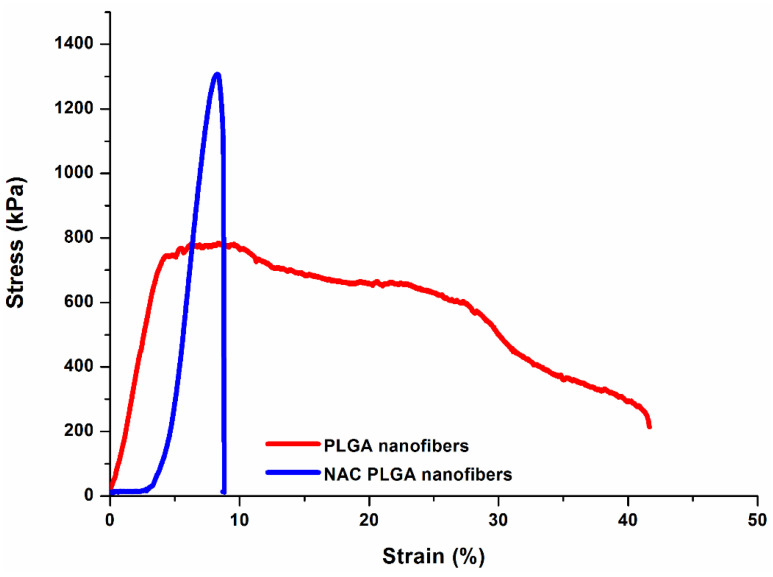
Stress–strain curves of the PLGA and NAC/PLGA nanofiber matrices obtained under a test speed of 0.167 mm/s.

Yield stress is vital to the translational success when choosing a material for structural purposes such as a scaffold. As a support structure, the scaffold needs to be robust enough to structurally accommodate tissue repair needs (hold cellular weight, fluid turbulence, adhering proteins, withstand remodeling) and the implantation process. Simultaneously, it must possess an elastic modulus similar to that of neural tissue and have the ability to encourage neuronal viability and extension [51]. The tensile yield stress of the NAC-loaded PLGA nanofibers was noted at 1294 kPa, which was significantly higher than that of PLGA nanofibers (765 kPa). The addition of NAC to the polymer matrix significantly modulates the stress–strain relationships for the nanofiber matrix (Figure 6). The NAC/PLGA curve exhibited a short toe region extending out to about 3% strain, followed by a linear rise in stress with strain and a sharp drop of the curve at 8.4% strain after nanofibers reached their tensile maximum stress. This implies that the majority of the NAC/PLGA nanofibers break simultaneously at the maximum load. Therefore, the tensile strength of the nanofiber mats may be approximately estimated as the sum of the tensile strength of all individual nanofibers that are subjected to strain during the test. In comparison, an extended plastic-like deformation region characterized by irreversible failure with progressive tearing and reduction in stress was noted for PLGA nanofibers only. The lack of adhesion between the polymer matrix due to dispersed NAC may be responsible for the early sharp drop of the curve observed for NAC/PLGA nanofibers as opposed to the extended deformation observed with unloaded PLGA nanofibers. PC12 neuronal cells have been previously noted to grow and spread on substrates as with a Youngs modulus of 184 kP [52]. The Youngs moduli were noted at 199 and 378 kPa respectively for NAC-free and NAC-loaded PLGA nanofibers. 

### 3.6. Analysis of NAC-Loading, Encapsulation, and Cumulative NAC Release 

**Figure 7:**
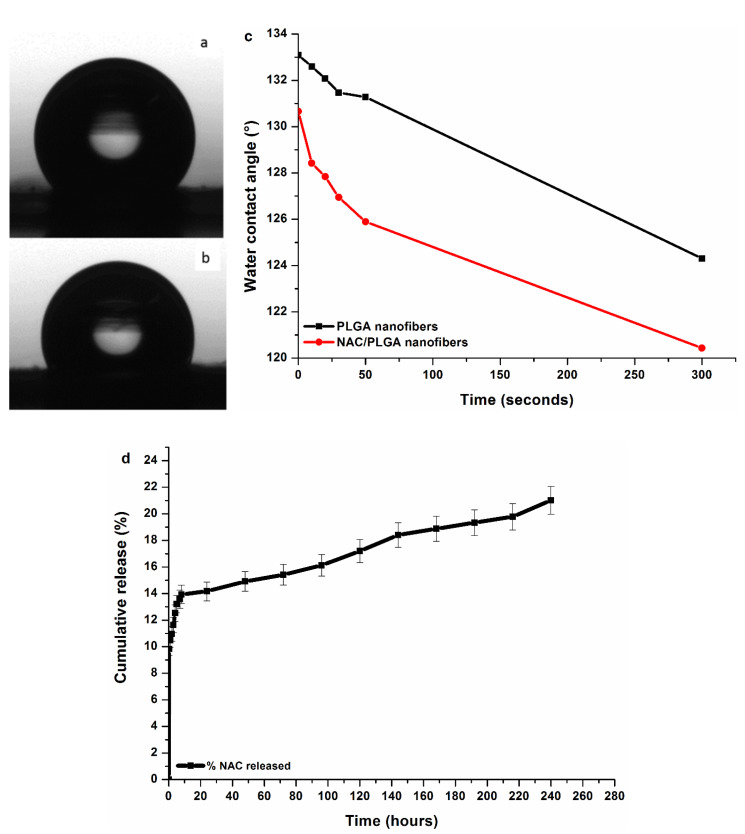
Water contact angle images of (**a**) NAC-free nanofibers and (**b**) NAC-loaded nanofibers (**c**) and changes within a 5-min period. (**d**) In vitro drug release (percent cumulative drug released) profile of NAC from PLGA nanofibers in PBS at 37 °C observed over 10 days (240 h).

A NAC encapsulation efficiency (DEE) value of 84% was calculated for the NAC-loaded PLGA nanofibers at 28% NAC loading. This high DEE can be attributed to the method of nanofiber formation, where electrospinning of the NAC polymer solution ensures that the NAC is entrapped within the nanofibers during fiber formation, and therefore, the NAC is embedded within the solid nanofibers. The hydrophilicity of the nanofibers determined by water contact angle measurement can actively influence the extent to which water diffuses through the scaffold (Figure 7). In turn, this will influence the hydrolysis of the nanofibers and release kinetics of the NAC released from the scaffold. In order to achieve a controllable delivery of NAC for efficient targeted localized delivery, we produced a delivery system based on electrospun fibrous poly(lactide-coglycolide) (PLGA), with a 85:15 lactide-to-glycolide ratio. The lactide portion is hydrophobic in comparison to the glycolide portion, and it makes up the larger ratio. Contact angles at < 90° indicate high wettability, whereas angles >90° indicate lower wettability [53]. As shown in Figure 7a–c, the water contact angles of both PLGA and NAC/PLGA are > 90°, indicating the low wettability of the nanofibers. N-acetylcysteine is a hydrophilic drug (e.g., carboxyl groups), and its improvement on hydrophilicity is observed (Figure 7). However, this is largely limited because PLGA was the major/main component of the scaffold and therefore, the hydrophobic properties of PLGA dominated (contact angle remained above >90). This means that the rate of water diffusion into the delivery system will be slow. This is desirable, as it is expected to aid in prolonging the release of the entrapped drug in the localized implanted area of the nanofiber scaffold. N-Acetylcysteine release from nanofibers is typically a biphasic process [54]. This process comprises of (1) burst release and (2) slower release rate [54,55,56]. The drug release profiles and characteristics of NAC/PLGA nanofibers are presented in Figure 7d. The plot of the cumulative NAC release as a function of time at 37 °C in PBS (pH: 7.4) shows biphasic drug release kinetics. The first stage, burst release, is characterized with an initial burst release during the first 8 h (13.9% cumulative release), where the rate of NAC release is very high in the initial 15 min and decreased considerably until 8 h. This is the initial release of surface associated drug molecules concurrently with fast diffusion into the aqueous phase. The initial burst release of NAC-loaded nanofibers resulted from the presence of phase separation of NAC particles on the fiber surface during the electrospinning process. The second stage was observed after 8 h with release of the remaining drug in a slow and stable manner. This is a solid-state diffusion of the drug from within the solid nanofibers (controlled slower release rate), which corroborates with the amorphization of the drug within the polymer phase observed in the XRD pattern. A percentage cumulative release of 21.0% was noted at 240 h (10 days) (Figure 7).

Following brain injury, reactive oxygen species (ROS) and reactive nitrogen species (RNS) are produced, which leads to cell and tissue death (via damage of proteins, nucleic acid, and lipids, and the depletion of endogenous antioxidants) [57]. However, the secondary injury of TBI evolves over hours and days, which provides a window of opportunity for intervention [58]. Clinical trials have noted the short half-life of antioxidants due to their oxidation as an inhibitory limitation. Therefore, entrapping the drug within the polymer matrix not only protects the drug, it also improves shelf life and provides localized delivery, circumventing systemic side effects. From a pharmaceutical perspective, drug release observed herein in the form of an initial burst may replenish endogenous antioxidant activity via glutathione up-regulation, while the subsequent controlled release offers sustained release of this antioxidant over a period of time during a critical intervention period (the first few hours to days prior to secondary injury evolution). Glutathion (GSH) is used by cellular enzymes to detoxify reactive oxygen and nitrogen species and to repair oxidatively damaged proteins; however, GSH cannot be taken up by neurons, but NAC can restore GSH levels by serving as a cell-permeable source of cysteine, which is a substrate for GSH synthesis [25]. Clark and co-workers conducted a randomized, double-blind Phase I study following TBI using NAC and probenecid, and they noted NAC concentrations ranging from 269.3 ± 113.0 to 467.9 ± 262.7 ng/mL in cerebrospinal fluid (CSF) at 24 to 72 h post-bolus, respectively [21]. Detection of these NAC drug amounts revealed no adverse effects following the surveillance of serum brain injury biomarkers attributable to drug treatment [21]. In this study, embedding the NAC-eluting PLGA nanofibers within the scaffold is proposed to potentially result in NAC accumulation within the scaffold matrix at the injury site from the biphasic release profile observed, which may potentially further result in neuroprotection at the injury site. The nanofibers are effective in acting as a drug delivery system.

### 3.7. Effect of NAC-Loading on Cell Viability and Proliferation 

**Figure 8:**
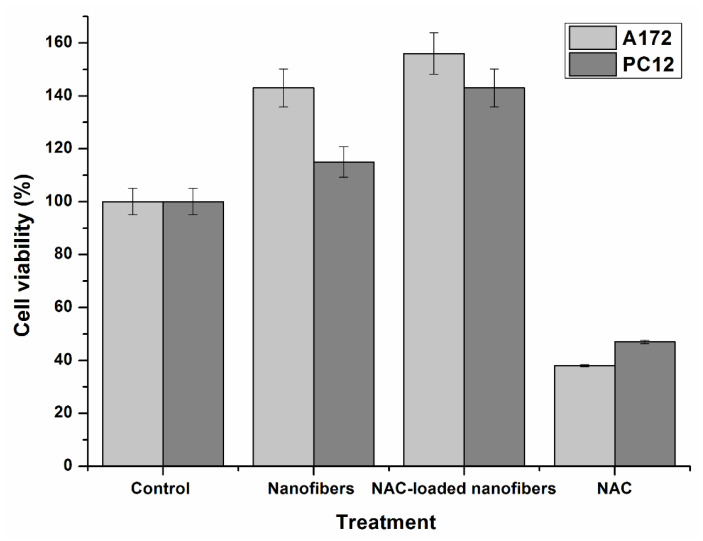
Cell viability and proliferation on control, and scaffold treatment of (**a**) PC12 and (**b**) A172 cell lines after 72 h incubation. Indirect cytotoxicity evaluation of various treatments based on the viability of PC12 and A172 cell lines that were cultured for 72 h.

Figure 8 shows the indirect cytotoxicity evaluation of the electrospun fiber mats based on the viability of PC12 and A172 cell lines that were cultured for 72 h. The percentage viability of the cells was reported as the percentage with respect to that of the control (media). In accordance with ISO 10993-5, cytotoxicity is determined as follows: cell viability above 80% is considered non-cytotoxic, cell viability within 80–60% is considered weak, 60–40% indicates moderate cytotoxicity, and below 40% indicates strong cytotoxicity, respectively [59]. 

According to ISO 10993-5, the PLGA and NAC-loaded PLGA nanofibers were non-toxic to both PC12 and A172 cell lines, as their percentage viability values were well above 80% (Figure 8). This is indicative of the cytocompatility of these systems, as they did not release any toxic substances in the cell culture medium toward PC12 and A172 cell lines. This was expected, as the carefully selected polymers materials NAC and PLGA herein are FDA-approved and noted for their biocompatibility, biodegradability, and non-toxic properties [15,60,61,62]. 

A percentage cell viability higher than that of the control was observed with the nanofibers (Figure 8). This suggests that the nanofibrous scaffold further enhanced the growth of cells. The observed favorability toward the nanofibers compared to the control as a substrate by the cells could be attributed to their architecture. Nanofibers most closely mimic the ECM and provide a high surface area to volume ratio for cell attachment and proliferation. 

The percentage viability on NAC-loaded nanofibers was higher than that of the NAC-free nanofibers. Ramburrun also noted an increase in neuronal cell (PC12) proliferation compared to NAC-free poly(3-hyroxybutyric acid-co-3-hydroxyvaleric acid) (PHBV) blended with magnesium-oleate (MgOl) fibrous films ascribed to NAC incorporation into the nanofiber system [63]. This may be attributed in part to the hydrophilicity imparted by the NAC (SH and NH groups). By comparison, moderate to strong cytotoxicity was noted for both A172 and PC12 cell lines when treated with NAC only (Figure 8). Media comprising of NAC crystal as a solute may have impeded cell growth by the formation of a hypertonic media, thereby altering cell tonicity. When NAC is embedded in the nanofibers, the slow release rate of the NAC from the nanofiber system may favor cell proliferation as opposed to burst exposure to the bare concentrated drug crystals. Overall, the incorporation of NAC into the nanofiber system confers a synergistic interaction from the observed improvement in neuronal survival and proliferation in comparison to the NAC-free nanofibers and the control. 

## 4. Conclusions

Biomimetic NAC-loaded PLGA nanofibers were produced via electrospinning. The presence, interactions between the native PLGA and the scaffold, and chemical integrity and performance of the nanosystem were confirmed by detailed physicochemical and physicomechanical studies. Scanning electron microscopy revealed the formation of a 3D nanofibrous structure with interconnected pores to support nutrient, oxygen, and cell migration as well as enable integrate surrounding cells into the scaffold within the ECM of brain tissue. In vitro release experiments of NAC demonstrated a biphasic release profile comprising an initial phase of 13.9% release within 8 h followed by more sustained release for a further 240 h. This is ideal for the nanosystem to function as a reservoir and elute the neuroprotective NAC at a TBI lesion. Ex vivo studies confirmed that the NAC-loaded nanosystem was non-toxic toward PC-12 and A172 cells with a positive effect on cell proliferation. Overall, the results showed that the nanosystem has promising potential for simultaneous application in both neural tissue repair and as a neuroprotectant in a spatial and temporal manner from a biomimetic neuroscaffold.

## Figures and Tables

**Table 1:** Parameters and settings employed during degassing and analysis of the nanofibers.

Sample Tube	Value
Warm free space:	1.0000 cm^3^
Cold free space:	1.0000 cm^3^
Non-ideality factor:	0.0000620
Use isothermal jacket:	Yes
Use filler rod:	Yes
Vacuum seal type:	Seal Frit
**Analysis Conditions**	
Preparation	
Fast evacuation:	No
Unrestricted evacuation from:	5.0 mmHg
Vacuum set point:	10 µmHg
Evacuation time:	0.10 h
Leak test:	No
Use Trans Seal:	No
**Free Space**	
Free-space type:	Measured
Lower dewar for evacuation:	Yes
Evacuation time:	0.10 h
Outgas test:	No
**Po and Temperature**	
Po and T type:	Measure Po at intervals during analysis. Calculate the Analysis Bath Temperature from these values.
Measurement interval:	120 min
**Dosing**	
Use first pressure fixed dose:	No
Use maximum volume increment:	No
Target tolerance:	5.0% or 5.000 mmHg
Low pressure dosing:	No
**Equilibration**	
Equilibration time (P/Po = 1.000000000):	20 s
Minimum equilibration delay at P/Po > = 0.995:	600 s
**Sample Backfill**	
Backfill at start of analysis:	Yes
Backfill at end of analysis:	Yes
Backfill gas:	N2
**Adsorptive Properties**	
Adsorptive:	Nitrogen @ 77.35 K
Maximum manifold pressure:	925.00 mmHg
Non-ideality factor:	0.0000620
Density conversion factor:	0.0015468
Therm. tran. hard-sphere diameter:	3.860 Å
Molecular cross-sectional area:	0.162 nm²
**Inside diameter of sample tube:**	9.53 mm

**Table 2:** Surface area and porosity criteria.

Surface Area	Pore Size
Very low surface area < 0.01 m^2^·g^−1^	Micropores < 2 nm [37]
Low surface area < 10 m^2^·g^−1^	Mesopores 2–50 nm [37]
High surface area > 250 m^2^·g^−1^	Macropores > 50 nm [37]
